# Incipient Resistance of *Helicoverpa punctigera* to the Cry2Ab Bt Toxin in Bollgard II® Cotton

**DOI:** 10.1371/journal.pone.0012567

**Published:** 2010-09-07

**Authors:** Sharon Downes, Tracey Parker, Rod Mahon

**Affiliations:** 1 CSIRO Entomology, ACRI, Narrabri, Australia; 2 CSIRO Entomology, Canberra, Australia; University of Sydney, Australia

## Abstract

Combinations of dissimilar insecticidal proteins (“pyramids”) within transgenic plants are predicted to delay the evolution of pest resistance for significantly longer than crops expressing a single transgene. Field-evolved resistance to *Bacillus thuringiensis* (Bt) transgenic crops has been reported for first generation, single-toxin varieties and the Cry1 class of proteins. Our five year data set shows a significant exponential increase in the frequency of alleles conferring Cry2Ab resistance in Australian field populations of *Helicoverpa punctigera* since the adoption of a second generation, two-toxin Bt cotton expressing this insecticidal protein. Furthermore, the frequency of *cry2Ab* resistance alleles in populations from cropping areas is 8-fold higher than that found for populations from non-cropping regions. This report of field evolved resistance to a protein in a dual-toxin Bt-crop has precisely fulfilled the intended function of monitoring for resistance; namely, to provide an early warning of increases in frequencies that may lead to potential failures of the transgenic technology. Furthermore, it demonstrates that pyramids are not ‘bullet proof’ and that rapid evolution to Bt toxins in the Cry2 class is possible.

## Introduction

Transgenic crops expressing toxins from *Bacillus thuringiensis* (Bt) revolutionized agriculture by providing opportunities for pest control with reduced reliance on insecticide sprays. The first generation Bt crops express single toxins and are predicted to be particularly vulnerable to resistance if the targeted pests are not initially highly sensitive to the deployed toxin [1]. There have been four recent claims of field-evolved resistance to proteins in single toxin Bt crops, all within the Cry1 class [2]–[5]. Theoretical models suggest that “pyramiding” or combining two dissimilar insect toxin genes will delay resistance more effectively than single-toxin plants, even if different single-toxin plants were deployed sequentially or in mosaics or seed mixtures [6]. Hence, pyramids are predicted to cause a great delay in the evolution of resistance [1].

The first generation of Bt-cotton available worldwide was Bollgard® (known as Ingard® in Australia), which expresses a titre of Cry1Ac toxin that declines with plant age [7], [8]. While Bollgard® provides excellent season-long control of the key cotton pests *Heliothis virescens* and *Pectinophora gossypiella*, post-flowering it permits survival of *Helicoverpa* species [9], [10]. In Australia Bollgard®was replaced in 2004/05 with Bollgard II®, which contains Cry2Ab plus the original Cry1Ac event in a pyramid, while in the USA both the single and two-Bt cottons have been grown since 2003. Although Bollgard II®is expected to provide excellent season-long control of the key cotton pests worldwide, including *Helicoverpa*, for at least one generation annually, Australian populations of *H. armigera* and *H. punctigera* may be exposed to toxic levels of Cry2Ab only [9].

Since 2004/05, Bollgard II® has comprised at least 80% of the cotton grown in Australia. The main targets are the bollworms, *Helicoverpa punctigera* and *H*. *armigera*. These species frequently co-exist on intensively sprayed crops but while *H. armigera* rapidly develops resistance to insecticide sprays, there is only one such report for *H. punctigera* despite its being physiologically and biochemically capable [11]. In cropping regions, *H. armigera* populations are characterized by spring recruitment of adults from overwintering pupae within the region [12]. In contrast, *H. punctigera* populations typically peak in spring, driven by large-scale migration into the region from inland [12]. It is thought that any resistance genes present in the small *H. punctigera* population resident in the cropping area are periodically swamped by susceptible individuals emigrating from the unsprayed (and thus unselected) inland refugia, thereby retarding the development of resistance [12].

We have monitored the frequencies of rare Bt resistance alleles in *Helicoverpa* spp. in Australian field populations exposed to Bt-cotton using F_2_ screens since 2002/03 [13]–[15]. The method employed generates isofemale lines that produce a proportion of individuals that are homozygous for recessive haplotypes present in their field-derived parents [16]. It can provide robust estimates of actual gene frequencies in field populations, and increases detected are evidence of evolution of resistance [16], but is not always used because of the laborious requirements associated with individual paired matings and assay of large groups of F_2_ progeny families [17]. Initially our program focussed on *H. armigera* as it was perceived to represent the major resistance threat, but in 2004/05, *H. punctigera* was incorporated more comprehensively upon detecting an allele conferring resistance to Cry2Ab. F_2_ screens performed with *H. punctigera* from regions that grow Bt-cotton (hereafter “cropping areas”) until 2006/07 isolated three *cry2Ab* resistance alleles and estimated their field frequency at 0.0018 (n = 2192 alleles, 95% Credibility Interval [CI] = 0.0005, 0.0040)[15].

Herein we contribute data obtained using F_2_ screens in 2007/08 and 2008/09 to our previously published frequencies for *H. punctigera* from cropping areas to demonstrate a statistically significant rise in the frequency of *cry2Ab* resistance alleles that appears to be increasing exponentially. This increase is mirrored by data from F_1_ screens performed on the same populations, initiated in 2007/08 and continued in 2008/09, which show a 3.2-fold rise in resistance frequency during the most recent season. Furthermore, F_1_ screen data demonstrate that in 2009 the frequency of *cry2Ab* resistance alleles in field populations of *H. punctigera* from inland Australia with no history of growing Bt-cotton or other crops (“non-cropping areas”) was 8-fold lower than that found in 2008/09 for populations in cropping areas.

## Results

### *cry2Ab* resistance allele frequency shifts in cropping areas estimated by F_2_ screens

During 2007/08, five of 286 isofemale lines (1144 alleles) from cropping area populations examined through F_2_ screens for Cry2Ab resistance scored positive, yielding an estimated *r* (resistance allele) frequency of 0.005 with a 95% CI between 0.002 and 0.010. During 2008/09, 11 of 251 isofemale lines (1004 alleles) examined through F_2_ screens for Cry2Ab resistance scored positive, yielding an estimated *r* frequency of 0.012 with a 95% CI between 0.006 and 0.020. The estimated *r* frequency of Cry2Ab in 2008/09 is 2.4-fold higher than, and exceeds the upper 95% CI, for 2007/08.

**Figure 1:**
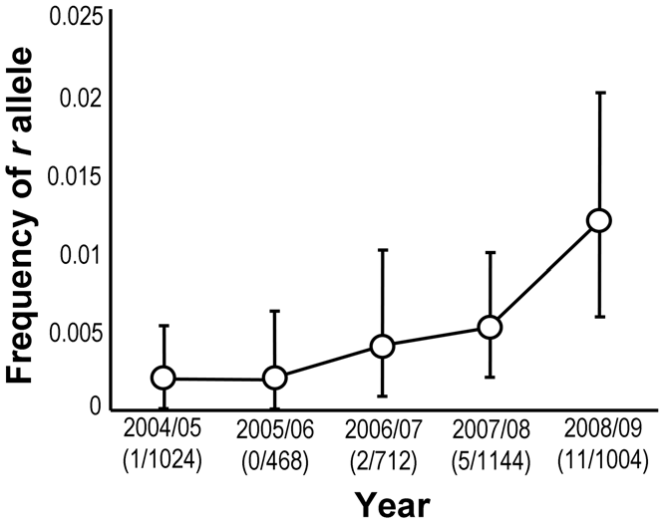
Frequencies of *cry2Ab* resistance alleles in *H. punctigera* from cropping populations. The values in parentheses below the years show the number of resistance alleles/the number of alleles tested. The data were collected using F_2_ screens.

A goodness-of-fit test on F_2_ screen data collected from 2004/05 to 2008/09 shows that the estimated *r* frequency of Cry2Ab varies significantly among seasons (χ^2^ = 15.4, df = 4, P = 0.004; Fig. 1). A simple linear regression demonstrates a significant positive relationship between the estimated *r* frequency of Cry2Ab and season (r^2^ = 0.80, P = 0.039). An exponential growth curve fitted to the same data shows a stronger positive relationship (r^2^ = 0.94; P<0.0001) which is largely driven by the 2008/09 data.

### *cry2Ab* resistance allele frequency shifts in cropping areas estimated by F_1_ screens

The increase observed for F_2_ screens is mirrored by data from F_1_ screens conducted on the same populations, for 2007/08 and 2008/09. These screens used testers from a Cry2Ab homozygous resistant colony (designated Hp4-13) established from a positive F_2_ screen in 2004/05. During 2007/08, two of 98 isofemale lines (196 alleles) from cropping populations examined for Hp4-13-like resistance to Cry2Ab scored positive, yielding an estimated *r* frequency of 0.015 with a 95% CI between 0.003 and 0.036. During 2008/09, 30 of 320 isofemale lines (640 alleles) from cropping populations examined for Hp4-13-like resistance to Cry2Ab scored positive, yielding an estimated *r* frequency of 0.048 with a 95% CI between 0.033 and 0.065. The estimated *r* frequency in 2008/09 is 3.2-fold higher than, and exceeds the upper 95% CI, for 2007/08. A Fisher's Exact test shows that the incidence of positive tests in the two years is significantly different (P = 0.016).

### Frequencies of *cry2Ab* resistance alleles in cropping vs. non-cropping populations

We also performed F_1_ screens of *H. punctigera* field populations from non-cropping areas and compared the frequencies with those for populations in cropping areas. In the non-cropping populations sampled in May 2009, five of 472 isofemale lines (944 alleles) examined for Hp4-13-like resistance to Cry2Ab scored positive yielding an estimated *r* frequency of 0.006 with a 95% CI between 0.002 and 0.012. The *r* frequency for cropping populations sampled in 2008/09 is 8-fold higher than for non-cropping populations sampled in 2009 and exceeds the upper 95% CI. A Fisher's Exact test shows that the incidence of positive tests between cropping and non-cropping populations is significantly different (P<0.0001).

## Discussion

Herein we provide two forms of evidence that evolution of resistance by a pest species to an insecticidal protein in dual-toxin Bt cotton has occurred: 1) significantly greater frequencies of resistance alleles in field populations from areas growing Bt-cotton versus areas that do not grow Bt-cotton; and 2) significant increases in the frequency of resistance alleles in populations from cropping areas since the widespread adoption of Bt-cotton. Our frequencies are from robust screens designed to detect rare resistance alleles [16]. The screens were performed across seasons in the same laboratory using a single protocol and toxin source and samples from the same range of hosts and dates. In all cases, the probability of detecting a resistance allele, if it indeed was present in sampled individuals, was at least 98%.

Most previous cases of field-evolved insect resistance to Bt crops involve plants producing only single toxins [18]. This report of resistance evolution in field populations is to a protein in a dual toxin Bt-crop. It is important to point out that at current frequencies of Cry2Ab resistance, field failures (i.e., complete inability to control a pest outbreak) are not observed. Since resistance is recessive [19], *rr* homozygotes must become markedly more common for field-failures to occur, and until 2008/09 no *rr* individuals were detected in *H. punctigera*. However, this is not surprising as at the current frequency of the resistant allele, we can expect homozygotes to be quite rare (expected frequency (0.048)^2^ = 0.002) if the population is in Hardy Weinberg equilibrium. If the exponential rise in frequency continues at the current rate, we expect that detectable numbers of homozygotes will become apparent in the next few years.

Most previous cases of field-evolved insect resistance to Bt crops involve toxins in the Cry1A class [18]. This is among the first published reports of field-evolved resistance to a Bt protein in the Cry2 class although several studies established baseline measures for future monitoring [13], [15], [20]. In particular, Ali and Luttrell [20] used dose-response assays to demonstrate that *H. zea* and *H. virescens* from different hosts and collections made at different times during the year have different susceptibilities to Cry2Ab but conclude that empirical data is needed to more clearly associate the variation to insect inheritance, field selection and other influences. However, analysis by Tabashnik et al. [21] of data reported by Ali and Luttrell [20] shows that the percentage of Cry2Ab-resistant *H. zea* populations increased from 0 in 2002 (the year before introduction of cotton plants producing Cry2Ab) to 50 in 2005. The resistance detected by Tabashnik et al. [21] was based on changes in the concentration of Cry2Ab that were lethal to 50% of the larvae tested (LC_50_). The susceptibility of key lepidopteran pests to Cry2Ab is highly relevant since Bt toxins in the Cry2 class are key events in current (and likely future) pyramided transgenic crops, and cross-resistance is likely to exist among specific toxins in this class [22].

The “high-dose plus refuge” strategy assumes that resistance to Bt is recessive and any homozygous resistant insects emerging from Bt crops are more likely to mate with the much larger number of susceptible insects emerging from non-Bt refuge crops than with each other. The offspring of such matings will be heterozygous and thus functionally susceptible to a high-dose of Bt toxin [23]. The strategy was adopted for Bt-cotton in Australia despite the target pests having relatively low inherent susceptibility to Cry1A and Cry2A toxins [13], [15]. Thus, it is generally accepted that the toxin concentration in Bt cotton may not always be “high” for Australian cotton pests, or indeed other *Helicoverpa* spp. worldwide [9], [13], [15]. Actually, on occasions the titre of both toxins must decline below lethal levels as in the three seasons from 2005/06 to 2007/08 an average of 15% of the area planted to Bollgard II® carried medium-large *Helicoverpa* spp. larvae at a rate exceeding 1 larvae per meter of cotton row [23]. In three fields affected in 2008, 0.2, 0.3, and 0.4 individuals per meter of cotton row survived to pupate on Bollgard II® and emerged as healthy moths, even though no genetic increase in resistance has occurred [24]. Importantly, despite Bollgard II® having two Bt insecticidal proteins, the expression of Cry1Ac probably consistently declines so that Cry2Ab is the only effective toxin late in the season [7], [8]. Thus, *rr* homozygotes in the field, like the Hp4-13 strain which is still genetically susceptible to Cry1Ac [19], may survive on Bollgard II® due to their Cry2Ab resistance alone. Changes in the Bt-cotton deployment strategy in Australia may therefore paradoxically increase the carryover of resistance to subsequent generations. When single-toxin Ingard® cotton was grown in Australia it was capped at 30% of the total cotton crop [25], and insecticidal sprays were required to control any larvae later in the season [9]. In contrast, there is no cap on the area that can be planted to Bollgard II®, and no control of potentially Bt-resistant larvae late in the season with insecticide sprays [26].

Previously we proposed that an agent other than Bt-cotton may favour selection for alleles conferring resistance to Cry2Ab in Australian *Helicoverpa* species [13], [15]. This notion is supported by this current report of populations of *H. punctigera* from non-cropping inland Australia carrying Hp4-13-like resistance alleles. However, an alternative hypothesis is that gene flow from cropping to non-cropping areas increased the frequency of *cry2Ab* resistance alleles in non-cropping populations. It is difficult to test for back-migration of *H. punctigera* from cropping areas to the inland because this presumably gradual process would be difficult to detect as shifts in population sizes, and no appropriate markers exist to track individuals. However, during autumn the appropriate wind patterns for transport are typically much weaker than those available for movement towards cropping regions in the spring, thus it seems unlikely that many moths could travel the 1000 km or more back to the inland.

Mutation-selection equilibrium theory predicts that if a mutation introduces resistance alleles and there is weak selection against them (as would be the case if there was a low fitness cost), a non-zero equilibrium resistance allele frequency can occur in the absence of selection favouring the resistance alleles [27]. Thus, it is also possible that random mutation, rather than selection by another agent, generated *cry2Ab* resistance alleles in Australian *Helicoverpa* populations before the introduction of cotton producing the Cry2Ab protein, and that this mutation is present in inland populations of *H. punctigera*.

If another agent in the environment selects for Cry2Ab resistance, to drive the shift in frequencies we have observed, the hypothesised agent must have increased in efficacy within cropping regions at the same time that Bollgard II® was introduced, without doing so in non-cropping areas. This coincidence seems unlikely. The resistance frequencies of populations from cropping areas are significantly greater than those of populations from non-cropping areas. Moreover, the current resistance frequencies in non-cropping populations (0.006) are perhaps similar to those predicted for cropping populations before opportunities for significant selection by Bollgard II® (0.008: See Materials and Methods). These sources of evidence strongly suggest that selection by the Cry2Ab toxin expressed in Bollgard II®, and not by another agent, is responsible for the increasing frequencies of resistance alleles in cropping populations.

Clearly, populations of *H. punctigera* that migrate from inland areas into cropping areas may carry *cry2Ab* resistance alleles, albeit at a lower frequency than presently exists in the cropping region. However, in the absence of quantitative information it is difficult to predict the dilution effect that migrating populations will exert on populations present in cropping regions. Long-term, pheromone trap records from the cropping region near Narrabri, N.S.W. show that the abundance of *H. punctigera* in the first generation (spring) is highly variable between years [28]. This pattern suggests that recruitment of moths to cropping regions from inland areas is erratic.

Additionally, there may be recent shifts in the tendency of *Helicoverpa* spp. to enter a facultative pupal diapause during winter in response to declining temperatures and photoperiod in southern growing areas. Studies conducted during the 1980's and 1990's found virtually no overwintering *H. punctigera* pupae [29], but this species comprised 47% and 21% of emerged moths from diapausing pupae in 2007 (n = 19) and 2008 (n = 100) [28]. Thus, perhaps in recent years *H. punctigera* has become more sedentary in cropping regions, and thus exposed to sustained selection for resistance.

The current situation is one in which monitoring for resistance has precisely fulfilled its intended function; namely, to provide an early warning of increases in frequencies that may lead to potential failures of the transgenic technology. Potential strategies that may mitigate further increases in resistance frequencies include imposing a cap on the area of Bollgard II® cotton, mandating larger dedicated refuges, and applying chemical insecticides to crops late in the season. Replacing Bollgard II® with cotton that expresses a novel insecticidal toxin that kills Cry2Ab resistant larvae could also significantly hinder the further evolution of Cry2Ab resistance. The most immediate challenge for the Australian cotton industry is to identify which of these possible changes to the current resistance management plan for Bollgard II® are feasible and appropriate for current frequencies of Cry2Ab resistance.

## Materials and Methods

### General Rearing

*H. punctigera* were maintained using the general rearing methods described previously [15], [19].

### Laboratory Strains

For the F_1_ screens, we used the *H. punctigera* Cry2Ab homozygous resistant colony Hp4-13 established from a positive F_2_ test in 2004. This strain has been repeatedly outcrossed to a susceptible colony and reselected with Cry2Ab toxin at a concentration that allows only homozygous resistant individuals to thrive. The resistance present in Hp4-13 is due to a single autosomal gene, is fully recessive, and homozygotes survive the highest concentration of Cry2Ab toxin that could be administered (500 µg/ml) [19]. We attempted to establish colonies whenever a *cry2Ab* resistance allele was isolated using F_2_ screens; the 6 additional colonies tested thus far are allelic with Hp4-13 and exhibit the same resistance characteristics.

Details of the susceptible colony, LHP, employed in this study are described previously [15], [19]. LHP was tested during every assay to verify that a correctly administered discriminating concentration was applied.

### Field collections

*H. punctigera* from cropping areas were sampled each season from October until April from a range of cultivated and uncultivated hosts in all of the major cotton growing areas in eastern Australia. The locations of these growing areas are given on the map in Downes et al. [15]. Most samples were collected as one egg per leaf to reduce the possibility of testing more than one individual from the same female parent. Material was randomly assigned to F_2_ screens or F_1_ screens.

*H. punctigera* from non-cropping areas were sampled in May 2009 from locations near Bedourie, Monkira, Cacoory Bore, Eyre Creek, and Mt Howitt in south-western Queensland, Australia. The nearest region with a history of growing Bt cotton (Emerald, Queensland) was at least 1,000 km from the nearest sampled location (Mt Howitt). Within each location, four main hosts (*Psorelea cinerea*, *Crasepedia* spp., *Calotis multicaulis*, *Sida* spp.) were sampled for larvae ranging from 2^nd^ to 5^th^ instar using a sweep net.

### F_2_ screen and F_1_ screen methods

F_2_ screen data for Bt-selected *H. punctigera* populations from 2002/03 until 2006/07 were reported previously [15]. Herein we used the same methods to expand this data set to include frequencies of *cry2Ab* resistance alleles for this species from cropping populations sampled in 2007/08 and 2008/09. In 2007/08 we began F_1_ screens with *H. punctigera* using Hp4-13. Herein we also report frequencies of *cry2Ab* resistance alleles for cropping populations of this species obtained using F_1_ screens in 2007/08 and 2008/09. We contrast these F_1_ data from 2008/09 with those obtained using the same method from non-cropping populations sampled in 2009. All of the screens were conducted in our Narrabri laboratory as part of the Bt resistance monitoring program supported by the Australian Cotton Industry [14].

The F_2_ screen and F_1_ screen bioassays used to identify resistant insects were conducted using published protocols [12]. Briefly, the assays were conducted in 45 well (2.7 cm^2^) trays which contained rearing diet that was overlaid with a suspension of toxin at a concentration of 1 µg/cm^2^ delivered in a 50 µl/well solution. Dried and ground corn leaf material provided by Monsanto Company (St Louis, MO, USA) was used as a source of Cry2Ab toxin. After the addition of one neonate larvae per well, trays were heat sealed and maintained at 25°±2°C and 45–55% RH. After 7 days, the larvae were scored as being “alive” or “dead”, and the growth stage (instar) of all survivors was recorded.

### Statistical analysis

Details of the Bayesian inference statistical approaches used herein, including the equations used to determine expected frequency of resistance alleles in the sampled populations (E[*p_R_*]) and the 95% credibility intervals for our estimated frequencies, are provided in Downes et al. [15].

Using the methods developed in Wenes et al. [30] we determined for populations from cropping areas that in 2007/08 and 2008/09 the joint 95% credible region for E[*p_R_*] from the F_1_ screen and F_2_ screen is statistically different and the probability that the two estimates are the same is <0.0001. For both data sets it therefore was not appropriate to combine data from the different screens to calculate an E[*p_R_*] that was representative of that season. Consequently we examine and report trends in data from F_2_ screens and F_1_ screens separately for populations from cropping areas.

F_2_ screens and F_1_ screens of the same population should yield similar frequencies of the target resistance allele. An exception might occur if F_2_ screens isolated more than one type of resistance gene but if so, they would yield higher, not lower, frequencies than F_1_ screens. F_1_ screens with *H. armigera* also yield higher frequencies of the target *cry2Ab* resistance alleles than do F_2_ screens of the same population [31]. Mahon et al. [31] disproved the hypothesis that some ‘resistance alleles’ (and possibly flanking regions) are homozygous lethal if autozygous (as generated in F_2_ tests) but not as allozygous homozygotes (as generated in F_1_ tests). Since the techniques we use ensure that it is difficult to overestimate frequencies, and a less complex method reduces opportunities for violating assumptions, we accept that F_1_ tests provide the more reliable estimate. However, note that our focus herein is on *relative* changes in frequencies, which are most relevant for indicating the development of resistance.

To ascertain the reliability of our screens we followed the methods developed by Andow and Alstad [16], Stodola and Andow [32], and Yue et al. [33] for calculating the probability of a false negative (*P_No_*) for a line in the F_1_ and F_2_ screens. For F_2_ screens and F_1_ screens the criteria used for each component of the calculation is identical to that outlined in Downes et al. [15] and Mahon et al. [31] respectively. In all cases, the probability that at least one of the lines was erroneously classified as susceptible was between 0.015–0.001. Thus, the probability that all possible resistance alleles in the parental lines were detected was between 0.985–0.999.

We examined variation over time of the incidence of positive tests using goodness-of-fit tests. We did not include data on populations from cropping areas collected in 2002/03 or 2003/04 that were published in Downes et al. [15] because this period was before *H. punctigera* was comprehensively incorporated into the monitoring program and only 2 and 12 isofemale lines respectively were screened.

Using data from F_2_ screens, we found that in 2004/05 before the widespread adoption of Bollgard II® the frequency of Cry2Ab resistance alleles in *H. punctigera* was 0.002 with a 95% CI ranging 0.0002 to 0.005 [15]. We did not perform F_1_ screens during this period. However, in both years that we have F_2_ and F_1_ screen data, the difference in frequencies is four-fold. Using this factor we can extrapolate back to when only F_2_ data are available to predict that before the widespread adoption of Bollgard II® the F_1_ screen frequency of Cry2Ab resistance genes in *H. punctigera* would have been 0.008.
